# Optimizing Waveform Maximum Determination for Specular Point Tracking in Airborne GNSS-R

**DOI:** 10.3390/s17081880

**Published:** 2017-08-16

**Authors:** Erwan Motte, Mehrez Zribi

**Affiliations:** CESBIO, Université de Toulouse, CNRS/CNES/IRD/UPS, 18 Avenue Edouard Belin, 31401 Toulouse CEDEX 9, France

**Keywords:** airborne, GNSS-R, land, processing

## Abstract

Airborne GNSS-R campaigns are crucial to the understanding of signal interactions with the Earth’s surface. As a consequence of the specific geometric configurations arising during measurements from aircraft, the reflected signals can be difficult to interpret under certain conditions like over strongly attenuating media such as forests, or when the reflected signal is contaminated by the direct signal. For these reasons, there are many cases where the reflectivity is overestimated, or a portion of the dataset has to be flagged as unusable. In this study we present techniques that have been developed to optimize the processing of airborne GNSS-R data, with the goal of improving its accuracy and robustness under non-optimal conditions. This approach is based on the detailed analysis of data produced by the instrument GLORI, which was recorded during an airborne campaign in the south west of France in June 2015. Our technique relies on the improved determination of reflected waveform peaks in the delay dimension, which is related to the loci of the signals contributed by the zone surrounding the specular point. It is shown that when developing techniques for the correct localization of waveform maxima under conditions of surfaces of low reflectivity, and/or contamination from the direct signal, it is possible to correct and extract values corresponding to the real reflectivity of the zone in the neighborhood of the specular point. This algorithm was applied to a reanalysis of the complete campaign dataset, following which the accuracy and sensitivity improved, and the usability of the dataset was improved by 30%.

## 1. Introduction

In the last thirty years, optical [1] and microwave [2,3] remote sensing approaches, based on active or passive techniques, have shown great potential to retrieve land surface parameters. In recent years, many studies have been carried out in an effort to analyze the potential of the Global Navigation Satellite Systems Reflectometry (GNSS-R) technique for the estimation of Earth surface parameters, in the fields of oceanographic and land-surface research [4]. These studies have shown that it is possible to retrieve various surface characteristics (soil moisture, biomass) from low-level observables such as Delay Doppler Maps (DDM) and complex waveforms. These developments have been achieved thanks to a large number of ground-based [5,6] and airborne [7,8,9,10,11,12] campaigns, during which the influence of various observational factors (elevation angle, polarization, etc.) was investigated. These measurements provided the first dataset that made the design and validation of dedicated bistatic GNSS scattering models possible [13,14,15,16]. Since the launch of TechDemosat-1 in 2014 [17] and the CyGNSS constellation in 2016 [18], there has been a shift in the sensitivity and potential retrieval of parameters such as soil moisture or vegetation cover, that can be achieved with spaceborne data [19,20]. Despite these crucial advances, since GNSS-R is a technique based on signals of opportunity (i.e., with which there is little or no control over their geometry, signal power or modulation), analysis of the acquired data is not as straightforward as in the case of other microwave instruments, which have been designed with the specific objective of Earth observation in mind. Filtering is generally applied to GNSS-R datasets, and more stringently to airborne datasets, in order to remove data acquired under non-optimal conditions, such as that derived from signals from low-elevation satellites, or during aircraft maneuvers such as takeoff, landing and turns [8,10]. In this context, the present study was designed to assess different approaches that could be used to improve the data processing, and to allow data to be retained for scientific analysis, which would otherwise have been deemed unusable as a consequence of noise or contaminating signals.

In the present paper we present a methodology designed to mitigate the effects arising from the use of GNSS-R receivers in aircraft. Section 2 briefly describes the GLORI airborne instrument, campaign dataset and processing scheme, whilst focusing on potential sources of error or signal corruption. In Section 3, we propose a technique for the improvement of waveform processing, which in particular allows signal maxima to be detected, and mitigates perturbations arising from the direct signal. Section 4 provides various examples produced using the proposed techniques, and suggests the use of several quantitative and qualitative improvement indicators. Finally, our conclusions are presented in Section 5.

## 2. The GLORI Instrument, Dataset and Processing Chain

### 2.1. Instrument

**Figure 1:**
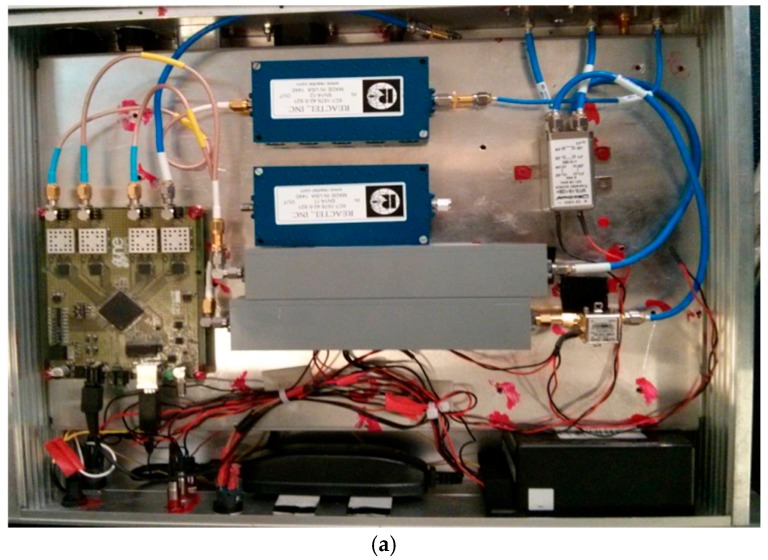

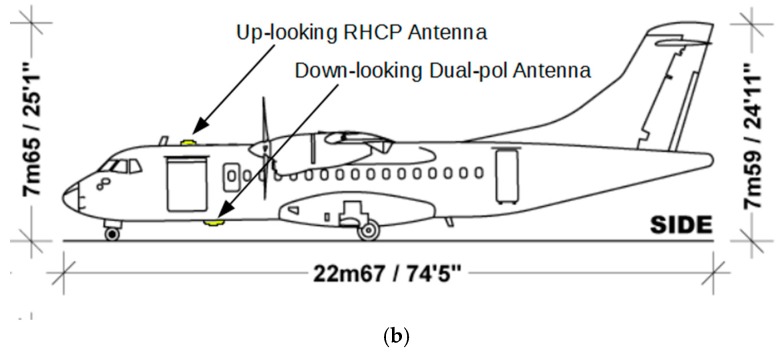
(**a**) The GLORI instrument; (**b**) The SAFIRE ATR 42 Aircraft.

GLORI is a GNSS-R polarimetric instrument [11] designed for aircraft operations. Two antennas are connected to the receiver: the first of these is an up-looking RHCP antenna used to monitor the direct signals transmitted by GNSS satellites, and the second is a down-looking, dual-polarization antenna (RHCP and LHCP) used to collect the signals reflected from the Earth’s surface. Both polarizations are cross-calibrated by means of a transfer switch. The nadir antenna is qualified in terms of isolation between the two LHCP and RHCP polarizations [11]. Figure 1 shows the instrument and the French ATR 42 aircraft on which it is mounted.

### 2.2. Airborne Campaigns

The GLORI airborne campaigns took place during the period from June to July 2015, and included a total of five flights over the south of France. Fifteen hours of data were acquired during these measurements, which involved three types of surface: agricultural areas, forest areas, and bodies of water. Simultaneously to these airborne measurements, various ground truth measurements were carried out in numerous test fields.

**Figure 2:**
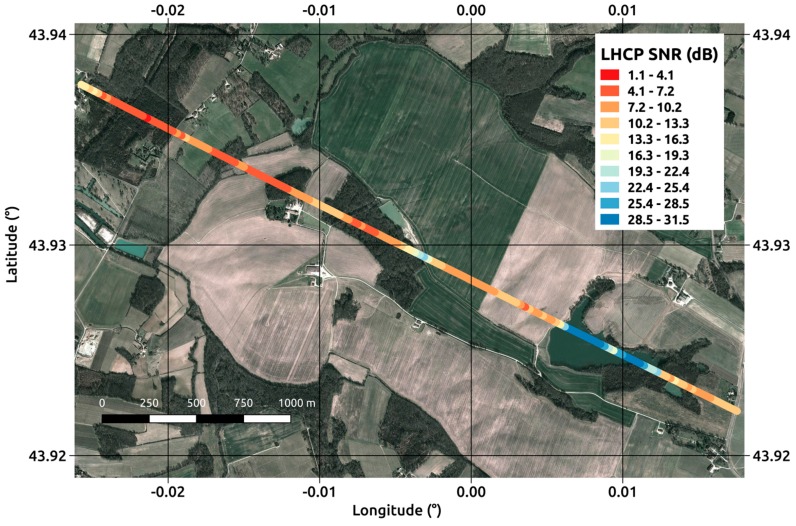
Reflected LHCP Signal to Noise Ratio for a 36-s transect acquired on the 22 June 2015. PRN23, Elevation angle 78°. The aircraft altitude is ~2000 m AGL, and the measurement temporal resolution (incoherent averaging time) is 240 ms.

These included measurements of soil parameters such as moisture and roughness and vegetation parameters such as Leaf Area Index (LAI), water content, height or Above Ground Biomass (AGB). Figure 2 provides a view of a measurement transect showing the reflected LHCP signals characterized by substantially different signatures, depending on whether they are recorded over water, agricultural soil or forest areas.

### 2.3. Processing Chain

**Figure 3:**
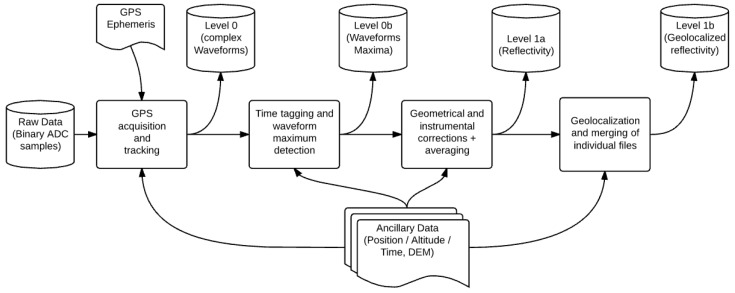
GLORI processing chain block diagram.

The GLORI processing chain (Figure 3) is composed of four main blocks using raw datastream, GPS ephemeris and flight ancillary data as inputs [11]:

1. GNSS processing of raw datastream
2. Acquisition and tracking of modulated signal to compute correlation waveforms
3. Time tagging and extraction of waveform maxima
4. Processing of the navigation message to get transmission time and extraction of the correlation power
5. Instrument corrections and incoherent averaging
6. Correction for antenna gain and instrumental noise, incoherent averaging and computation of reflectivity
7. Geolocalisation and merging of individual files
8. Computation of the footprint location and shape on the surface, merging of individual measurements in a consolidated file

### 2.4. Focus on the Estimation of Waveform Maxima

The GLORI instrument has a 10 MHz sampling rate, allowing a temporal resolution of 0.1 μs to be achieved. In the case of a direct signal or specular reflection, the width of the GPS C/A code correlation waveform is 1.955 μs (2 × *T_chip_* = 2 × 1 ms/1023) where *T_chip_* is the duration of one code chip. The shortest window length required to resolve the full waveform is thus greater than 20 samples. However, in order to account for any possible window misalignment, and to resolve any possible waveform broadening resulting from scattering by rough media, a window size of 61 samples (±30) was selected for the processing of GLORI data.

In order to center the correlation window over the reflected area, a geometric delay needs to be estimated. In the present case, this is computed from flight parameters and added to the delay determined for the direct signal:

(1) $$\Delta \tau_{model}=2hsin\left(\theta \right)f_{s}/c,$$

where *h* is the aircraft’s height above ground level (AGL), *θ* is the satellite elevation angle, *f_s_* is the receiver sampling frequency defining the waveform resolution in the delay dimension, and *c* is the speed of light.

In the GLORI processing chain, the delay of the reflected signal can be adjusted only per blocks of 8 samples (~0.8 chips), which add a potential bias of ±3 samples with respect to the correlation window center. When the uncertainties in aircraft altitude, satellite azimuth and elevation angle (corresponding to the specular point location and corresponding terrain elevation) are added, the position uncertainty of the correlation window is found to be of the order of ±5–10 samples (0.5–1 μs). Finally, since the correlation delay is constant for each 36-s acquisition sequence, it cannot account for the changes in range associated with takeoffs, landings, flight altitude variations and terrain elevation variations.

For these reasons, apart from the correlation window centering, an additional system is needed to track any displacement of the waveform peak, since the correlation power at this point is related to the scattering contribution from the area surrounding the specular point. A crude approach would involve searching for the waveform maximum corresponding to each coherent integration period. Although this could appear to be a logical method in the case of specular surfaces associated with a high SNR (bodies of water for example), and high elevation angles, this method has two main limitations: (1) the weak reflected SNR for short integration times and/or areas of low reflectivity would make it difficult to detect the waveform peak; and (2) the possible contamination of reflected waveforms by direct signals in the case of low aircraft altitudes and/or low elevations, leading to an incorrect estimation of the peak position. To overcome these limitations, we compare several different approaches, as described below. The aim of this analysis is not only to reduce the errors in estimated waveform peak positions and related signal power, but also to allow a higher proportion of the data to be included in the scientific analyses.

**Figure 4:**
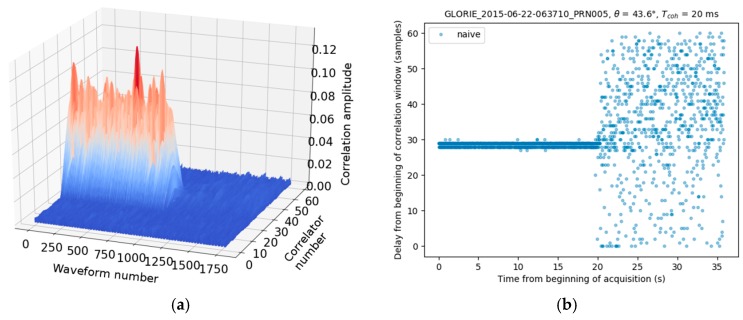
(**a**) Time series of Waveforms for a lake to forest transition; (**b**) Naive estimation (max) of the peak location. Aircraft height is ~650 m AGL.

In an initial phase, we consider a simple, “naive” approach involving the identification of maxima in the window of 61 lags. The location of this peak is estimated to coincide with that of the waveform maximum $\tau_{spec,n}\left(t\right)$:

(2) $$\tau_{spec,n}\left(t\right)=argmax\left(\left|Y\left(t,\tau \right)\right|\right),$$

where *argmax*() is the operator giving the location of the wafeform maximum in the delay dimension, and Y(t,τ) is the complex correlation waveform. Figure 4 illustrate this simple approach, when applied to the case a GLORI acquisition during a transition from a lake to forest-covered hills. Figure 4a shows the reflected waveform time series, during which the transition from water to forest is clearly revealed (after approximately 1000 waveforms) by a sudden fall in the correlation amplitude. Figure 4b shows the resulting impact on the delay, corresponding to the specular point reflection when using the aforementioned “naive” identification of waveform maxima. Over the forest, the naïve method only returns a random location of the peak maximum as the reflected waform signal power level is below the noise power level (SNR < 0).

## 3. Optimizing the Detection of Waveform Maxima

Using the extensive dataset provided by the GLORI 2015 airborne campaign, we analyzed the reflected waveforms and evaluated several different methods designed to improve the tracking of the waveform peak, corresponding to signal contributions generated by the area surrounding the specular point. In particular, we focused on signals recorded with short integration times and/or originating from low reflectivity surfaces, which could lead to a poor SNR, as well as those recorded under conditions of low elevation angles and/or altitudes, which could be contaminated by the direct signal.

Four approaches to the improvement of peak detection are described in this section:

- Smoothing of the peak locations determined with the naive method (NS)
- Incoherent averaging used to improve the SNR (IA)
- Incoherent averaging and smoothing (IAS)
- Implementation of an algorithm using the above methods, in addition to adaptive filtering over the delay range, designed to mitigate the direct signal contributions (DM)

We illustrate these methods through the use of various reflected waveform time series, corresponding to different surface conditions (water cover, bare soils, forest cover) and observation configurations (low and high elevation angles).

### 3.1. Smoothing with a Low-Pass Filter (NS)

As shown above, when dealing with low SNR reflected waveforms, a peak tracking system based on the detection of signal maxima does not work well, because the positions of the maxima are affected by random variations.

**Figure 5:**
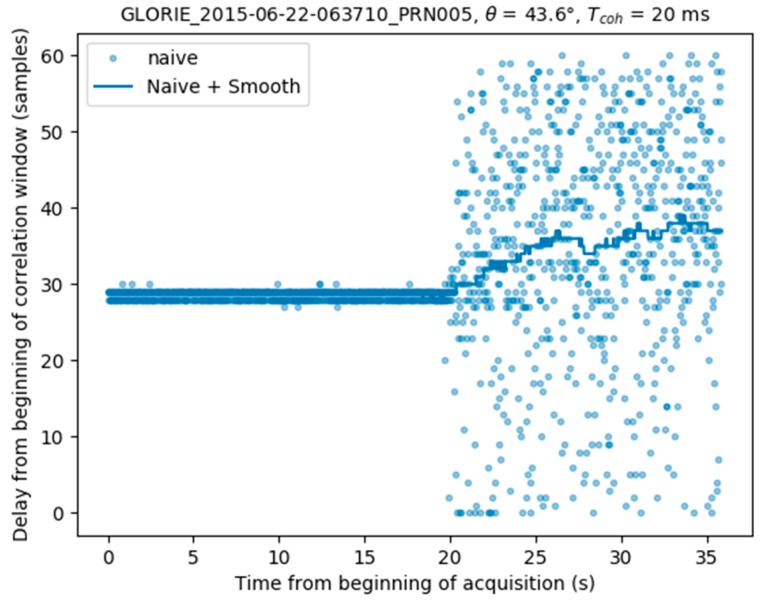
Estimation of the delay corresponding to the specular reflection location using a running-average smoothing.

Tracking of the peak can be improved through the use of a Savitsky-Golay filter [21], which acts like a moving-average low-pass filter. The rationale behind this approach is that the delay associated with the position of the specular point cannot vary more quickly than a simple function, which is directly related to variations in aircraft altitude, and to the topography of the overflown terrain. By selecting a filter width corresponding to 3 s of data (a horizontal distance of 300 m at the aircraft’s groundspeed of 100 m/s), fast variations in peak location were removed. The delay corresponding to the maximum of the peak $\tau_{spec,ns}\left(t\right)$ can be expressed as:

(3) $$\tau_{spec,ns}\left(t\right)=SG_{3s}\left(\tau_{spec,n}\left(t\right)\right),$$

where $SG_{3s}$() is the filtering operation. Figure 5 provides a plot of the computed peak locations (light blue) estimated using the naive approach: the delay remains stable while the aircraft overflies a lake, and then becomes very noisy (at *t* = 20 s) when it overflies the forest. The dark blue plot shows the smoothed (low-pass filtered) result, which appears to be considerably more realistic.

### 3.2. Incoherent Averaging (IA)

In some cases, the previous approach does not perform sufficiently well, as the reflected signal can be contaminated by the direct signal, thus biasing the apparent position of the maximum towards shorter delays. A different approach was thus tested, which involved incoherent averaging of the waveforms, in order to reduce the noise and improve the peak location $\tau_{spec,ia}\left(t\right)=$ estimations:

(4) $$\tau_{spec,ia}\left(t\right)=argmax\left(<|Y\left(t,\tau \right){|}^{2}>\right)$$

where < > represents the averaging along time. An incoherent averaging time similar to that of the GLORI reflectivity products was selected, i.e., 240 ms. This method usually performs well, and was able to constrain the maximum values between 3 and 4 samples in the case of horizontal flight paths over flat surfaces.

### 3.3. Incoherent Averaging and Smoothing (IAS)

The two previous methods can be combined in an effort to smooth the results obtained using the incoherent averaging technique. This leads to smoothed variations in the maximum location $\tau_{spec,is}\left(t\right)$:

(5) $$\tau_{spec,is}\left(t\right)=SG_{3s}\left(\tau_{spec,ia}\left(t\right)\right)$$

**Figure 6:**
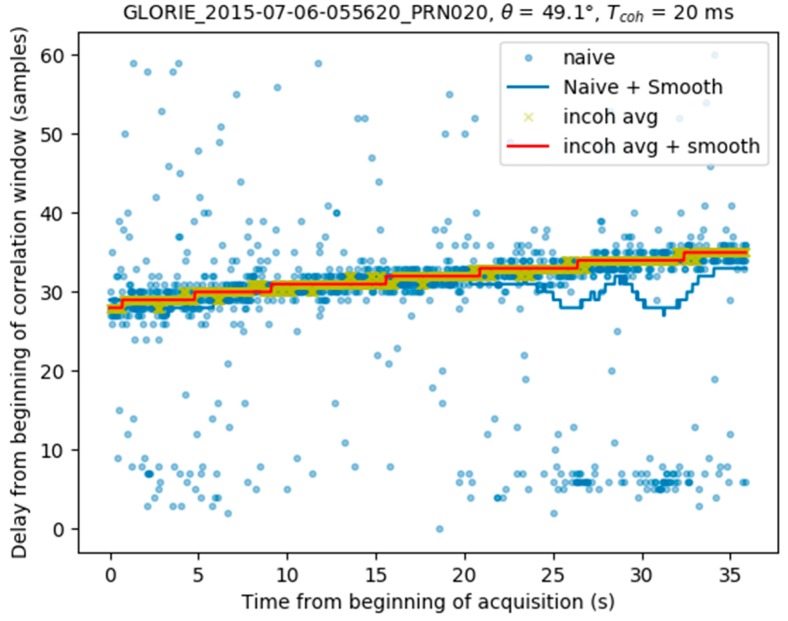
Peak detection using naïve, naïve + smoothing, incoherent averaging and incoherent averaging + smoothing methods. Aircraft height above ground level is ~500 m.

Figure 6 provides an example of data acquired during the takeoff phase of a flight, and processed using the methods described above. The naive peak detection technique (light blue dots) can be seen to be very noisy, whereas the peak loci shown by the dark blue line are smoothed. This figure reveals fluctuations towards the end of the acquisition, which are caused by a contribution from the direct signal visible from the naive approach. The difference in delay between the reflected and direct signal contributions (~25 samples) is in line with what is expected from a receiver flying at about 500 m above ground level, which is the case for the aquisition. The yellow crosses indicate the peak locations computed by incoherent averaging over 240 ms, whereas the red line is computed by smoothing these values with a low-pass filter. It can be seen that towards the end of the acquisition, the values estimated by incoherent averaging are not affected by fluctuations.

### 3.4. Direct Signal Mitigation Algorithm (DM)

**Figure 7:**
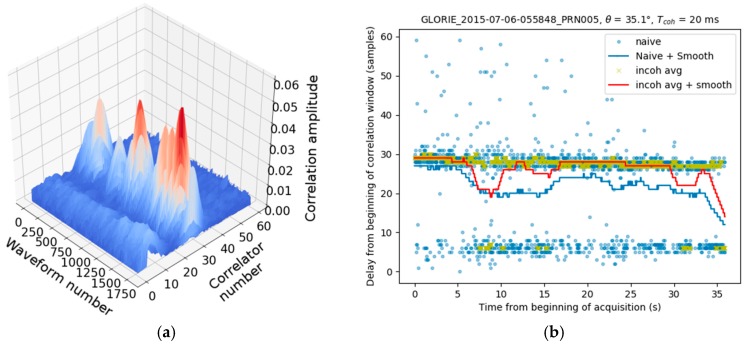
(**a**) Reflected waveform time series in case of strong direct signal contamination; (**b**) Peak detection using methods described before: It can be seen that both the naive and incoherently averaged solution are suffering from the direct signal peaking at five samples from the beginning of the correlation window. In both figures the aircraft height is ~590 m AGL.

Even when averaged and filtered, large errors can still be present in the computed loci of the waveform maxima, especially when the aircraft is flown at a low altitude, and/or when the GNSS satellites are at mainly low elevation angles. These errors can often be attributed to direct signal contamination of the reflected waveform. An example of this type of contamination, leading to errors in the peak detection algorithms, is shown in Figure 7.

**Figure 8:**
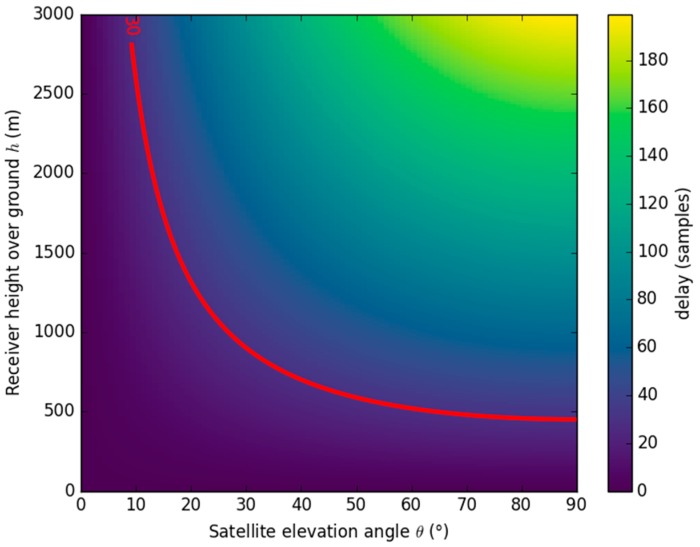
Delay between direct and reflected signals for various elevation angles and aircraft height. In the case of the current GLORI processing, all the area below the 30 sample red contour line can be subject to contamination by the direct signal.

Figure 8 shows the theoretical difference in delay between the direct and reflected signals, as a function of aircraft height AGL and GNSS satellite elevation angle. For a correctly centered correlation window of 61 lags, the reflected waveform could be contaminated by a difference in delay equal to ≤30 samples, in particular when a hemispherical antenna with strong side and back lobes is used, as in the case of the GLORI instrument, and during the turns. The red line on Figure 8 shows that this contamination can occur at any elevation angle during flights at an altitude below 500 m, and can place a limit on the minimum observation elevation angle at higher altitudes.

In order to mitigate the effects of direct signals leaking into the reflected signal antenna, we developed a technique designed to (i) detect the presence of a direct signal in the reflected waveforms; (ii) discard the contaminated delay bins in the peak search algorithm; (iii) implement the incoherent averaging and smoothing techniques described above.

**Figure 9:**
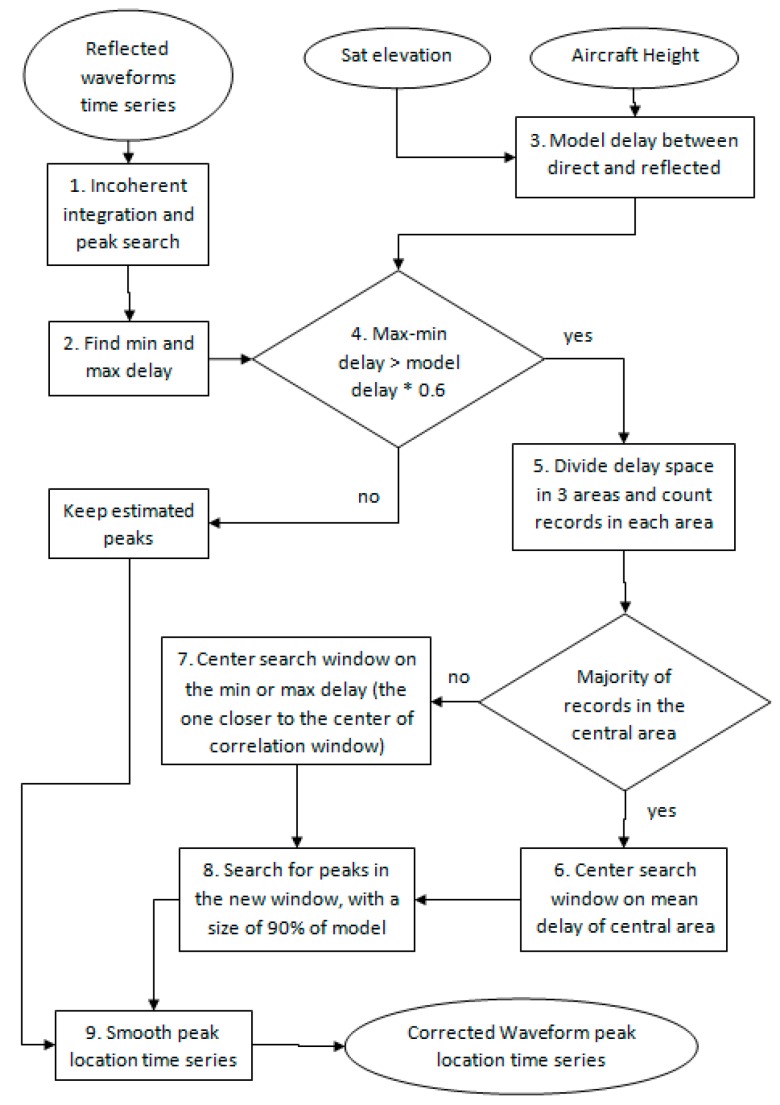
Flowchart of the multipath mitigation algorithm used for the GLORI data.

**Figure 10:**
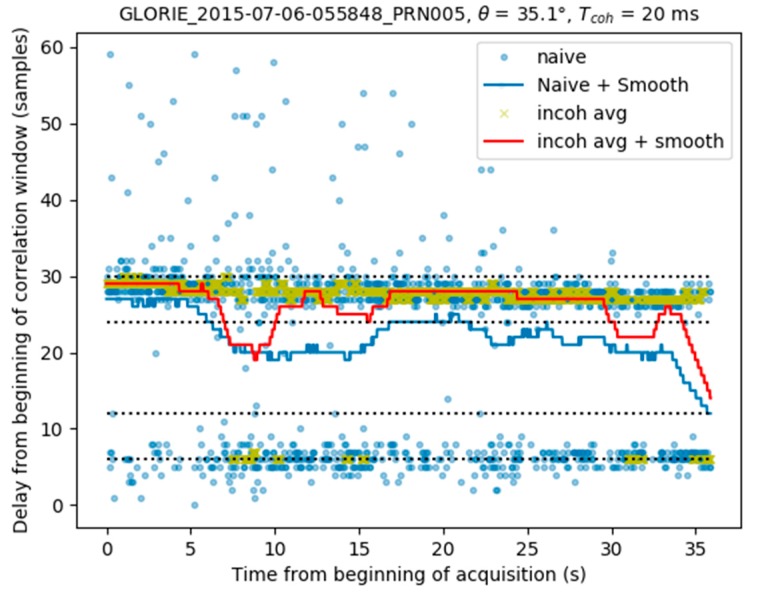
Partitioning of the delay space into three distinct zones, between the min and max values.

**Figure 11:**
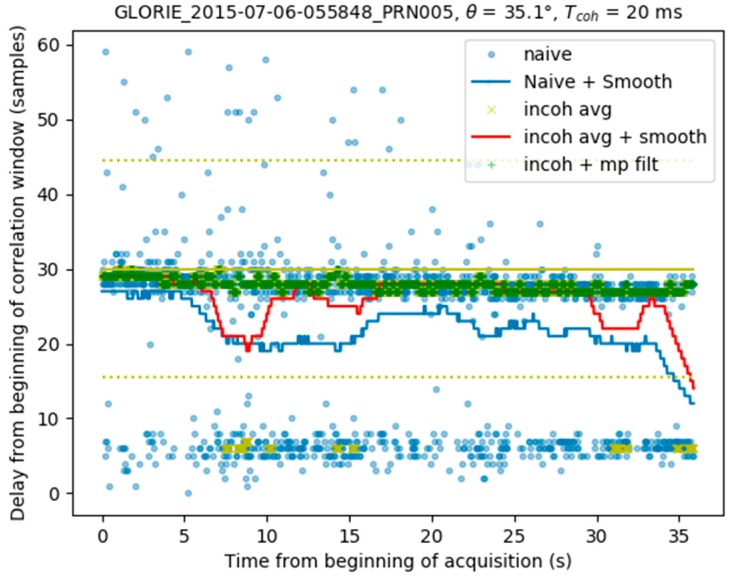
Peak search within the new window, after incoherent integration.

**Figure 12:**
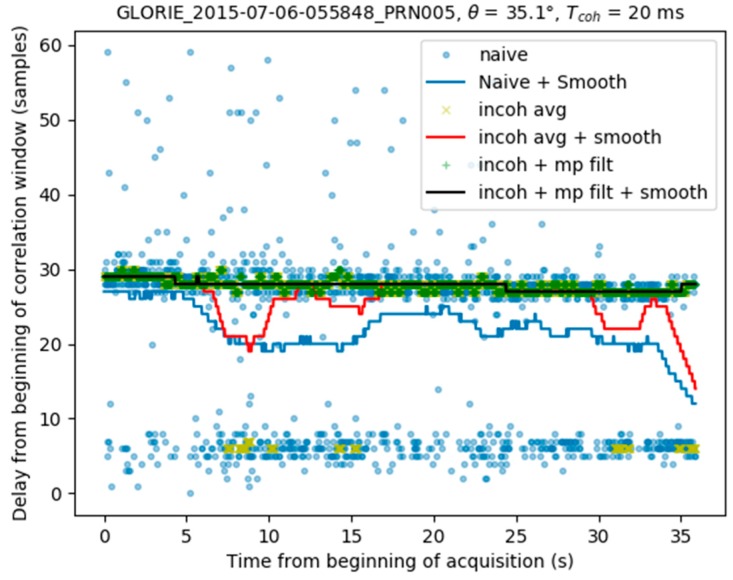
Smoothing of the direct signal mitigation algorithm result.

This algorithm is illustrated in Figure 9 and described in the following:

1. Following incoherent averaging without smoothing, a first guess is used to estimate the position of the maxima $\tau_{spec,ia}\left(t\right)$. (Equation (4))
2. The minimum $\tau_{spec,min}$ and maximum $\tau_{spec,max}$ values of the estimated delay are computed for the 36 seconds time series, together with their difference $\Delta \tau_{data}$:
  (6) $$\tau_{spec,min}=min\left(\tau_{spec,ia}\left(t\right)\right)\;\tau_{spec,max}=max\left(\tau_{spec,ia}\left(t\right)\right)\;\Delta \tau_{data}=\tau_{spec,max}-\tau_{spec,min}$$
3. The theoretical difference in delay between the direct and reflected signals $\Delta \tau_{model}$ is computed using (Equation (1))
4. If $\Delta \tau_{data}<0.6\times \Delta \tau_{model}$, the first estimation of the peak location $\tau_{spec,ia}\left(t\right)$ is retained, and no direct signal contamination is detected
5. Otherwise, the delay space is divided into three areas within the min-max delay range: 25% lower, 50% middle, 25% higher, in order to identify the location of most of the peaks (Figure 10)
6. If most of the peaks are located in the central zone, no direct signal contamination is identified, and the variations in delay estimation are attributed to ambient noise. A new peak search window is then created, centered on the average location of those peaks situated in the central zone
7. If most of the measurements are located in either the lower or higher delay window, the window having its mean value closest to the center of the correlation window (lag 31 in our case) is used as the new peak search window
8. A new peak search is carried out with incoherent averaging (see previous section) within the new peak search window, which has a total width equal to 90% of the theoretical difference in delay:
  (7) $$\tau_{spec,dm}\left(t\right)=argmax\left(Y\left(t,\tau \right)\right),$$
  where $\left|\tau -\tau_{center}\right|<0.45\times \Delta \tau_{model}$ and $\tau_{center}$ is the new location of the correlation window. In Figure 11 this window corresponds to the area between the two yellow dashed lines.
9. The final peak time series is smoothed, using the procedure described in Section 3.1. Figure 12 illustrates the final result, in which the peak locations determined by the full algorithm (black line) are found to be very stable, with no contamination from the direct signal.

**Figure 13:**
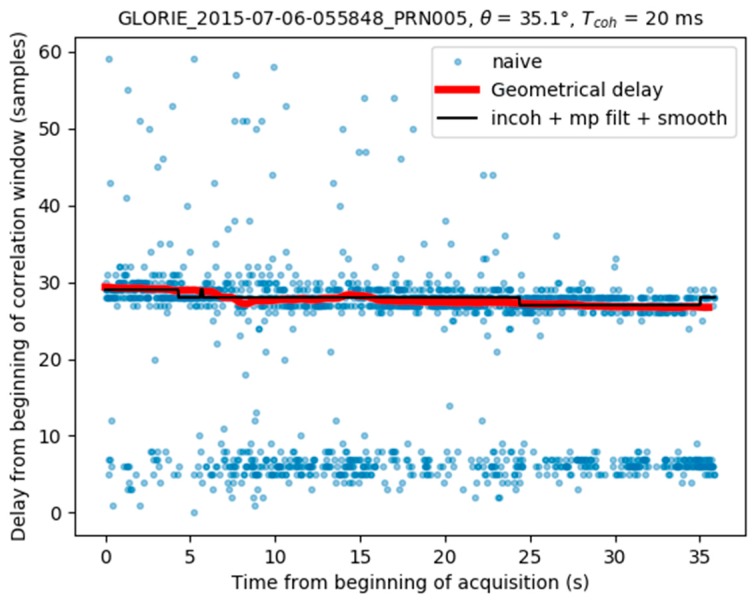
Comparison of the obtained peak location with the geometrical delay.

Finally, Figure 13 shows the estimated position of the waveform maximum compared to a geometrical delay computed from the aircraft height, the elevation at the specular point location and the elevation angle. It can be seen that the variation of the estimated delay is in line with the modeled geometrical delay.

## 4. Impact on the Dataset

### 4.1. Improvements in Signal Sensitivity

**Figure 14:**
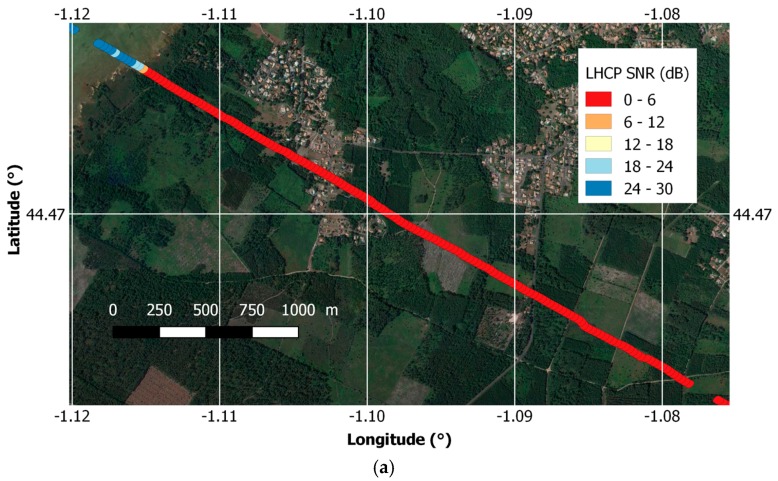

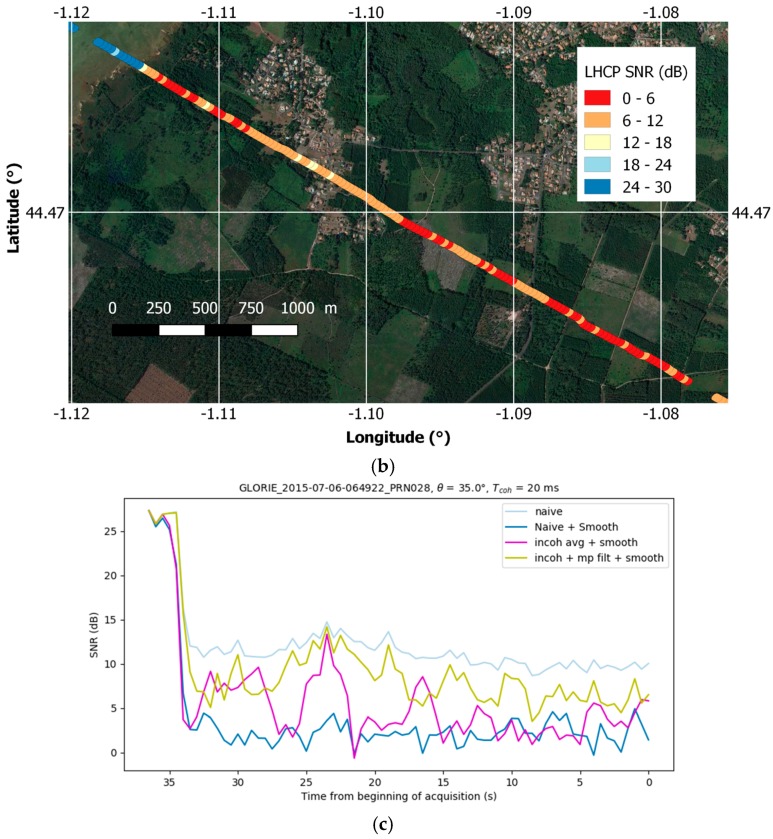
Reflected LHCP SNR for a transect over water and land for PRN28 at 35° elevation. (**a**) using naïve + smooth technique and overlaid with a google Earth Image; (**b**) using the new proposed approach and also overlaid with a google Earth image; (**c**) Comparison between the different methods.

Figure 14a,b shows an example for a transect corresponding to PRN28 (elevation 35°) covering water, fields, forests, and a small town, the color code from blue to red represents the reflected LHCP signal SNR superimposed over an image from Google Earth. The upper panel (a) shows the SNR computed using the naïve + smoothing approach which was used in the original GLORI processing chain. It can be seen that except over the water, the signal is saturating all the time with very low SNR value.

In Figure 14b, the same transect is shown using the new technique with direct signal mitigation. In that case, the dynamics is recovered and a sensitivity to difference in land use changes can be seen. Figure 14c shows the variations of SNR for this transect as computed by the different methods. It can be seen that the naïve approach is the one with less sensitivity, The proposed approach is improving this sensitivity, and the naive + smooth and incoherent averaging + smoothing are not able to properly reproduce the variations.

### 4.2. Improvements in Data Availability

**Figure 15:**
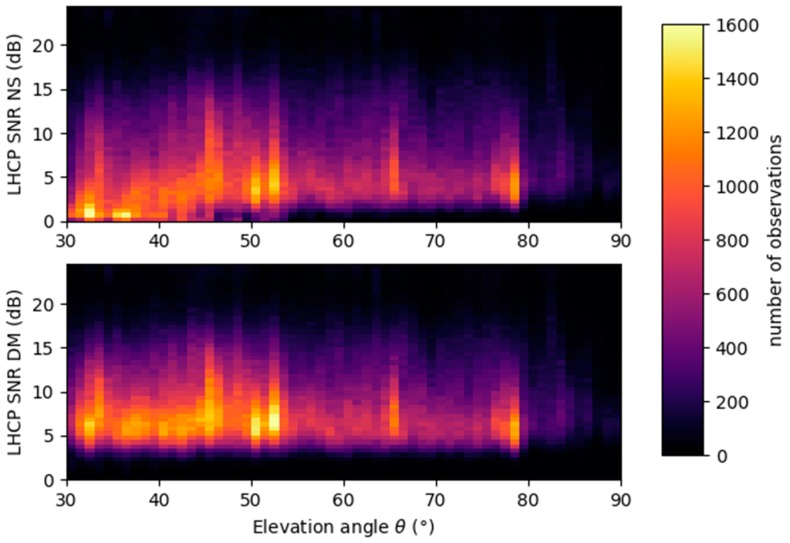
Data availability vs. SNR and elevation angle for original dataset (**top**) and reprocessed dataset (**bottom**).

Figure 15 shows the data availability vs. SNR (antenna gain corrected) and elevation angle for the whole GLORIE 2015 campaign. On the top panel, the values from the initial processing are shown, showing a rather constant distribution of the measurements vs. SNR at high elevation angle, and a drop towards low SNRs at elevation angles below 45°, proving a degradation of the maximum tracking method. For this reason, elevations angles below 45° where filtered out in the initial dataset. In the lower panel, the same data availability is shown for the dataset using the new direct mitigation algorithm. It can be seen that the distribution vs. SNR remains constant at all elevation angles, showing the good performance of the specular point tracking algorithm.

**Figure 16:**
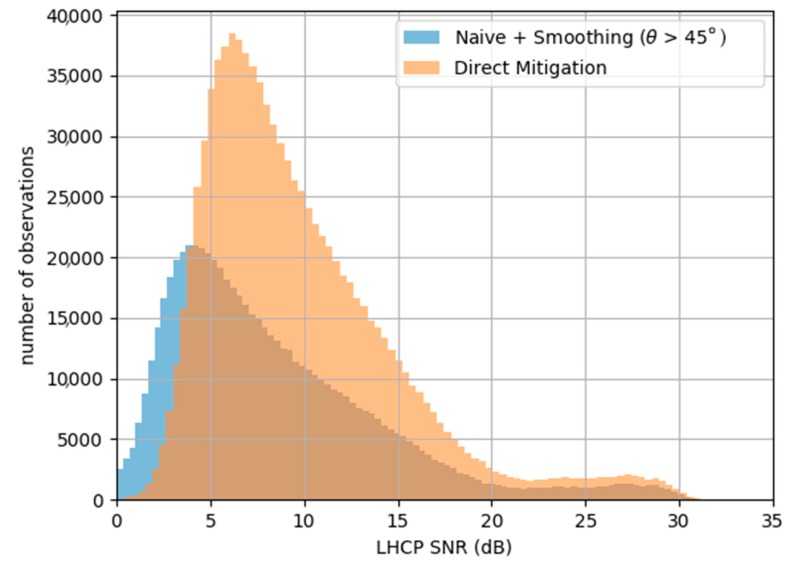
Data availability vs. SNR for the filtered original dataset (blue) and for the reprocessed dataset (orange).

Figure 16 is a histogram showing the distribution of the measurements versus SNR for the whole GLORI 2015 dataset. As stated above, in the original version of the dataset, measurements were filtered out below 45° of elevation to avoid SNR issues. Thanks to the direct mitigation algorithm, we avoid the SNR issues, and measurement from all elevations angles down to 30° can be kept. In total there are more than 955,000 individual 240 ms measurements that can be used with the new algorithm, when with the initial processing technique, only 633,000 were kept after filtering. This represents 33% of the dataset that was not used and that is now available.

## 5. Conclusions

We describe the importance of correctly detecting specular area contributions in noisy and direct-signal contaminated reflected waveforms. Following a description of the effects of low-pass filtering (Savitsky-Golay running average) and incoherent averaging on standard peak detection techniques, we propose an empirical algorithm designed to monitor the variations in delay resulting from changes in terrain or altitude during detection, and to remove possible contamination from the direct signal.

Our results reveal a significant enhancement in the accuracy with which signal contributions from the area surrounding the specular point (which is the basic observable used in land scatterometry applications) can be determined. This leads to improvements in terms of sensitivity, when compared to more simple approaches, and in terms of accuracy, when the direct signal is contaminating the waveforms.

We estimate that approximately 30% of additional airborne data, acquired at low altitudes, or from the signals transmitted by low-elevation (down to 30°) satellites, or during aircraft turns, could be improved by this technique.
